# At the heart of the problem: congestive cardiac failure as a cause of ascites: A narrative review

**DOI:** 10.1097/MD.0000000000029951

**Published:** 2022-08-05

**Authors:** Zhong Ning Leonard Goh, Roland Yii Lin Teo, Bui Khiong Chung, Alexis Ching Wong, Chen-June Seak

**Affiliations:** a Department of Medicine, Sarawak General Hospital, Ministry of Health, Kuching, Sarawak, Malaysia; b Department of Emergency Medicine, New Taipei Municipal Tucheng Hospital, New Taipei City, Taiwan; c Department of Emergency Medicine, Lin-Kou Medical Center, Chang Gung Memorial Hospital, Taoyuan, Taiwan; d College of Medicine, Chang Gung University, Taoyuan, Taiwan.

**Keywords:** cardiac ascites, cardiac cirrhosis, congestive cardiac failure, peritoneal fluid analysis, serum-to-ascites albumin gradient, ascitic protein, N-terminal pro-brain natriuretic peptide

## Abstract

Heart failure leading to cardiac ascites is an extremely rare and underrecognized entity in clinical practice. Recognizing cardiac ascites can be difficult, especially since patients presenting with ascites may have more than 1 etiology. Various biomarkers are available to aid in the diagnosis of cardiac ascites, though with differing sensitivities and specificities. Such biomarkers include serum albumin, ascitic albumin and protein, as well as serum N-terminal pro-brain natriuretic peptide (NT-proBNP). While serum NT-proBNP is a powerful biomarker in distinguishing the etiology of ascites and monitoring treatment progression, its cost can be prohibitive in low-resource settings. Clinicians practicing under these circumstances may opt to rely on other parameters to manage their patients. We go on further to report a series of 3 patients with cardiac ascites to illustrate how these biomarkers may be employed in the management of this patient population. Clinicians should always keep in mind the differential diagnosis of cardiac failure as a cause of ascites. The resolution of cardiac ascites may serve as a surrogate clinical marker for response to antifailure therapy in lieu of NT-proBNP at resource-scarce centers.

## 1. Introduction

Ascites refers to the accumulation of fluid in the peritoneal cavity. Liver cirrhosis and malignancy accounts for the vast majority (91%) of ascitic patients,^[1]^ though there exist lesser-known etiologies which are not adequately recognized by clinicians. One such example is heart failure leading to cardiac ascites.

Recognizing cardiac ascites can be difficult, especially since patients presenting with ascites may have more than 1 etiology.^[1]^ Various biomarkers are available to aid in the diagnosis of cardiac ascites, though with differing sensitivities and specificities. The basic laboratory investigations would invariably include liver function tests, renal profile, coagulation studies, and full blood counts.^[2,3]^ Further investigations include serum albumin, ascitic albumin and protein, as well as serum N-terminal pro-brain natriuretic peptide (NT-proBNP). While serum NT-proBNP is a powerful biomarker in distinguishing the etiology of ascites and monitoring treatment progression, its cost can be prohibitive in low-resource settings.

In this narrative review, we examine the available diagnostic modalities in the diagnosis and subsequent monitoring of patients with cardiac ascites. We go on further to report the clinical manifestation, treatment, and prognosis of a series of 3 patients with cardiac ascites to illustrate how these biomarkers may be employed in the management of this patient population.

## 2. Methods

Articles were retrieved from MEDLINE and EMBASE using the search terms “cardiac ascites”, “congestive cardiac failure”, “diagnosis”, “management”, and “biomarkers”. The search results were subsequently reviewed for relevance; all article types were included to synthesize this narrative review.

An illustrative case series was further obtained via a retrospective study at the Department of Internal Medicine, Sarawak General Hospital, Malaysia. The medical records of all patients diagnosed with cardiac ascites at Sarawak General Hospital from Jan 2020 to Aug 2020 were retrospectively extracted and reviewed.

This study protocol was approved by the Medical Research & Ethics Committee (MREC), Ministry of Health Malaysia (NMRR-20-1868-56120) in accordance with the Declaration of Helsinki, waiving the need for consent from study participants.

## 3. Discussion

Ascites is caused by a variety of underlying pathophysiologies, which can be broadly classified into portal hypertension, hypoalbuminemia, and peritoneal disease.^[4]^ Cardiac ascites in particular is an extremely rare entity which comprises 3% of all ascites. It is nevertheless important for clinicians to be aware of such a possibility, as some patients may have more than 1 cause of ascites.^[1]^

Cardiac ascites arises from right heart failure. Decompensated right ventricular failure results in an elevated right atrial pressure, which is then transmitted via the inferior vena cava and hepatic veins to the liver sinusoids, producing portal hypertension. The resultant sinusoidal congestion and enlarged fenestrae leads to exudation of protein-rich fluids into the space of Disse. These fluids are initially drained into the liver’s lymphatics; once the volume of exudate overwhelms the lymphatic system’s capacity, it is shunted into the peritoneal cavity and manifests clinically as ascites.^[5,6]^

Chronic passive venous congestion also impairs diffusion of oxygen and nutrients to hepatocytes, resulting in ischemia and liver fibrosis. This in turn further hampers the delivery of oxygen and nutrients, creating a vicious cycle with consequent worsening of ascites.^[5,6]^ Exacerbation of ascites may occur with the onset of splanchnic vasodilatation, renal hypoperfusion (causing overactivation of the renin-angiotensin-aldosterone system), and sodium retention leading to a hypervolemic state.^[7–9]^ The impact of cardiac cirrhosis on the patient’s overall prognosis has yet to be well established, hence its management is primarily treatment of the underlying cardiac disease.^[10]^

Differentiating between cardiac and liver cirrhosis requires analysis of the ascitic peritoneal fluid, in particular ascitic albumin and total protein levels. Serum-to-ascites albumin gradient (SAAG) is a useful indicator in identifying ascites caused by portal hypertension, with an accuracy of 80%–100%.^[11]^ Since both cardiac and hepatic etiologies cause ascites via portal hypertension, we expect SAAG to be >1.1 g/dL in both situations. Subsequent discrimination between the 2 etiologies would require an analysis of ascitic total protein, which is generally ≥2.5 g/dL in cardiac ascites.^[12]^ Such an employment of SAAG followed by ascitic total protein enables physicians to diagnose cardiac ascites with an accuracy of 95%.^[13]^ Further workup of echocardiogram should be done to assess for heart function and ejection fraction.^[14,15]^

Serum NT-proBNP is an established biomarker of heart failure used in clinical practice with numerous wide-ranging applications: to identify patients at risk of developing heart failure, confirm or exclude presence of heart failure, prognosticate patients diagnosed with heart failure, guide therapy in these patients, and even serve as end points in clinical trials.^[16]^ With upper limits of 300 pg/mL and 125 pg/mL in acute and nonacute settings respectively, NT-proBNP has a positive predictive value of 0.44–0.67 and negative predictive value of 0.94–0.98 for heart failure.^[17]^ A consensus has yet to be reached regarding its employment in outpatient follow-up. Nevertheless, some studies have found that NT-proBNP-guided therapy may be associated with better patient outcomes, especially all-cause mortality and cardiovascular hospitalization.^[18–20]^

One major disadvantage with this approach is that the cost of serial serum NT-proBNP investigations can prove prohibitive in resource-scarce centers with limited funding. Observations from our 3 patients suggest that the resolution of cardiac ascites and other symptoms may serve as a surrogate clinical marker for response to antifailure therapy in lieu of NT-proBNP.

### 3.1. Case 1

A 68-year old lady with underlying hypertension, diabetes mellitus, and dyslipidemia presented with a 2-week history of abdominal distension, dyspnea, and bilateral lower limb swelling, associated with constitutional symptoms. Physical examination was unremarkable save for gross ascites and bilateral pedal edema. Analysis of her peritoneal fluid revealed SAAG of 1.8 g/dL with ascitic protein of 2.5 g/dL, suggesting presence of portal hypertension. Subsequent echocardiogram demonstrated normal ejection fraction with a grade 2 diastolic dysfunction.

We proceeded with additional investigations—serum tumor markers, cytology studies of peritoneal fluid, upper and lower gastrointestinal scopes, and computed tomography scan of thorax, abdomen, and pelvis—to rule out malignancy in view of the presence of constitutional symptoms; all returned negative. N-terminal pro-brain natriuretic peptide (NT-proBNP) was later found to be 5255 pg/mL, supporting the diagnosis of cardiac ascites. The patient was initiated on diuretics and antifailure therapy, with eventual resolution of ascites. NT-proBNP on 1-month follow-up was reduced to 1780 pg/mL.

### 3.2. Case 2

A 75-year old lady with underlying hypertension and atrial fibrillation presented with a 3-month history of abdominal distension, associated with bilateral lower limb swelling and exertion dyspnea. On examination, there were bilateral crepitations and gross ascites. Echocardiogram demonstrated right heart dysfunction with normal ejection fraction. Peritoneal fluid analysis was suggestive of portal hypertension (SAAG 1.4 g/dL, ascitic protein 3.5 g/dL). NT-proBNP was >9000 pg/mL. The patient was given diuretics and heart failure treatment. Her ascites resolved in 2 weeks, with repeat NT-proBNP reduced to 5440 pg/mL.

### 3.3. Case 3

A 46-year old lady with bronchiectasis, pulmonary hypertension, and right heart failure presented with shortness of breath, orthopnea, bilateral leg swelling, and abdominal distension for 2 weeks. Examination likewise found gross ascites and bilateral lower limb swelling. Peritoneal fluid analysis revealed SAAG of 1.5 g/dL with ascitic protein of 3.4 g/dL. NT-pro BNP was 5558 pg/ml. The patient’s ascites was markedly reduced after initiation of heart failure treatment.

As previously mentioned, the management of cardiac ascites is primarily treatment of the underlying cardiac disease. The utility of such an approach is evident in our 3 patients—the initiation of diuretics and antifailure therapy brought about a quick and dramatic resolution of their ascites, as well as an improvement in NT-proBNP levels.

## 4. Conclusions

Although very rare, patients presenting with ascites may have underlying heart failure. Peritoneal fluid analysis suggestive of cardiac ascites (SAAG > 1.1 g/dL, ascitic protein ≥ 2.5 g/dL) should prompt further evaluation of echocardiogram and NT-proBNP to look for heart failure. Initiation of diuretics and antifailure therapy often produce dramatic resolution of ascites in such patients.

## Acknowledgments

We would like to thank the Director General of Health Malaysia for his permission to publish this article. The authors thank the staff of Sarawak General Hospital involved in the management of these patients.

## Author contributions

Conceptualisation: Goh ZNL, Chung BK, Seak CJ

Methodology: Goh ZNL, Chung BK, Seak CJ

Data curation: Goh ZNL, Teo RYL, Chung BK

Writing - original draft: Goh ZNL, Chung BK

Writing - review & editing: Goh ZNL, Wong AC, Seak CJ

Supervision: Goh ZNL, Chung BK, Seak CJ

Funding: Seak CJ.
